# Defining the tumor microenvironment of non‐small cell lung cancer

**DOI:** 10.1111/imcb.70114

**Published:** 2026-04-10

**Authors:** Kidane Siele Embaye, Tony Blick, Vahid Yaghoubi Naei, Ken O'Byrne, Brett G. M. Hughes, Arutha Kulasinghe

**Affiliations:** ^1^ Frazer Institute, Faculty of Health, Medicine and Behavioural Sciences The University of Queensland Brisbane QLD Australia; ^2^ School of Biomedical Engineering University of Technology Sydney Sydney NSW Australia; ^3^ The Princess Alexandra Hospital Brisbane QLD Australia; ^4^ The Royal Brisbane and Women's Hospital Brisbane QLD Australia; ^5^ Health, Medicine and Behavioural Sciences University of Queensland Brisbane QLD Australia; ^6^ Queensland Spatial Biology Centre Wesley Research Institute, The Wesley Hospital Brisbane QLD Australia

**Keywords:** immunotherapy, lung cancer, NSCLC, tumor microenvironment

## Abstract

Lung cancer is the most frequent cause of cancer‐related mortality globally. In recent years, the treatment landscape for advanced‐stage non‐small‐cell lung cancer (NSCLC) has been transformed by the advent of immunotherapy, which has yielded unprecedented and durable clinical responses for some patients. This shift has powered a significant expansion of novel immunotherapeutic strategies in oncology over the past few decades, heralding a new direction in the management of NSCLC. Despite these advances, only a fraction of patients acquire sustained benefit from immunotherapy, while the majority often develop resistance, resulting in disease progression leading to death. Several factors may contribute to this limited success, notably tumor heterogeneity and the intricate composition of the tumor microenvironment (TME). As a result, it is imperative to fully dissect the heterogeneity of the TME by identifying the principal drivers of cancer progression and immunotherapy resistance. This understanding is crucial for optimizing individualized treatment approaches. Here, we provide a summary of NSCLC, discuss the cardinal features of the TME and review advanced technologies such as spatial profiling of tissues, which are useful in dissecting the dynamicity of the tumor ecosystem. In addition, we highlight the recent developments and future perspectives of immunotherapy and other emerging therapeutics, as well as the role of predictive biomarker testing to ensure tailored therapy and tackle drug resistance, on the pathway to precision medicine.

## Graphical abstract

The tumor microenvironment (TME) in non‐small cell lung cancer is highly heterogeneous, both at a patient level and tumor microenvironment resolution. Defining the tumor microenvironment from patient‐responders and non‐responders will aid in the development of targeted therapeutics specific for each individual patient.

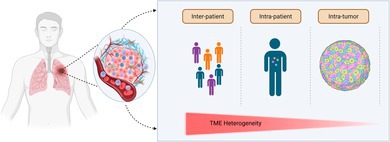

## INTRODUCTION

### Non‐small cell lung cancer

Based on the 2022 global cancer report, lung cancer remains the leading cause of cancer related mortality worldwide, with approximately 2.5 million new cases and over 1.8 million deaths.^1^ Non‐small cell lung cancer (NSCLC) makes up around 85% of all lung cancer cases and is further categorized into three main morphological subtypes: adenocarcinoma (50%), squamous cell carcinoma (35%) and large cell carcinoma (15%).^2^, ^3^ Small cell lung cancer (SCLC) is the second histological variant, which displays a more aggressive behavior with rapid proliferation and poor survival outcomes.^4^ Patients with advanced disease of these cancers originating from the mucosa of the bronchus or the gland‐forming structures have an unfavorable prognosis with less than one‐year median overall survival.^5^

Surgical excision of tumors, together with chemo‐radiotherapy, has traditionally been considered the backbone of cancer treatment. Starting from the early 1990s, platinum‐based chemotherapy has played a key role in the management of NSCLC and continues to serve as the standard first‐line therapy for metastatic disease.^6^ Nevertheless, diminished survival rates have been noticed in lung cancer patients owing to the failure of these conventional therapies to prevent progressive and recurrent disease.^7^ Over the past two decades, there has been a marked evolution in the therapeutic landscape of NSCLC, driven by the utilization of immune checkpoint inhibitors (ICIs). These agents work by inhibiting immune regulatory pathways such as programmed death ligand‐1 (PD‐L1)/programmed death‐1 (PD‐1) and cytotoxic T lymphocyte‐associated antigen‐4 (CTLA‐4), significantly improving survival outcomes in many patients.^8^, ^9^, ^10^, ^11^ Notably, the combination of PD‐L1/PD‐1 pathway and CTLA‐4 pathway inhibitors has demonstrated augmented therapeutic effectiveness, likely due to their synergistic interaction; however, this approach might be associated with a high rate of treatment‐related adverse effects.^11^

Under normal physiological states, immune checkpoints, along with co‐stimulatory molecules, maintain immune homeostasis by promoting self‐tolerance and modulating the immune response through either inhibitory or stimulatory signals.^12^, ^13^ In cancer settings, however, tumor cells exploit these pathways by disrupting checkpoint signaling, thereby escaping immune surveillance. Thus, ICIs were developed to counteract this evasion strategy by harnessing the individual's immune arsenal to destroy cancerous cells. The approval of these immunotherapeutic drugs by the US Food and Drug Administration (FDA) has marked a significant advancement in oncology, particularly in the context of advanced malignancies that have poor prognoses and limited treatment options. Despite their success, the use of ICIs in cancer immunotherapy has faced several limitations in clinical settings. First, only a subset of patients, typically 20%–30% of those with NSCLC and an even lesser number of patients with SCLC, show a meaningful response to single‐agent immunotherapy.^14^, ^15^ A high proportion of patients may not benefit from ICI therapy due to a lack of response after initial treatment (primary resistance) or their disease may progress, despite continued treatment, months after an objective response is observed (acquired resistance).^16^, ^17^, ^18^ Second, although immunotherapy offers clinical benefits, immune‐related adverse events occur in approximately 50–60% of patients, with 10–20% experiencing severe (grade 3–4) toxicities.^19^, ^20^, ^21^, ^22^, ^23^ These drug‐related adverse events, ranging from mild skin rash to fatal myocarditis, remain a major challenge in cancer care.^20^, ^21^, ^22^, ^23^ The incidence of life‐threatening events is rare (seen in ∼0.3–1% of patients), but treatment discontinuation may be required. Subsequent management often involves immunosuppressive medications, such as corticosteroids, to counteract the side effects of immunotherapy, which may lead to unwanted opportunistic infections.^24^, ^25^ Clearly, the concurrent reduction of inflammatory adverse events with preservation of immune‐based treatment represents a formidable challenge.

Although ICIs and molecularly targeted therapies have substantially improved the treatment landscape of NSCLC, their long‐term clinical efficacy is often compromised by the development of both primary and acquired resistance.^26^ As a result, a novel class of anticancer therapies called antibody‐drug conjugates (ADCs) has emerged, showing encouraging clinical benefits in NSCLC management.^27^ Mechanistically, ADCs are designed with the goal of boosting the antitumor function of monoclonal antibodies (mAb) by coupling them with cytotoxic agents. In essence, they are immunoconjugates composed of a tumor‐targeting mAb conjoined with a cytotoxic payload via a specialized linker molecule.^27^, ^28^ In 2022, trastuzumab‐deruxtecan (Enhertu) received accelerated FDA approval to treat HER2‐mutant NSCLCs, thereby paving the way for ADCs to be an integral part of the NSCLC treatment regimen.^29^ Recent research indicates that novel biomarkers may predict the efficacy and resistance of ADCs in NSCLC. Trophoblast cell‐surface antigen 2 (Trop2) and human epidermal growth factor receptor 3 (HER3) have recently been identified as promising ADC targets, with early‐phase clinical trials of agents like patritumab‐deruxtecan and datopotamab‐deruxtecan yielding promising results.^30^, ^31^ Additionally, elevated HER2 expression and gene mutations continue to serve as significant predictors of response to trastuzumab‐deruxtecan.^29^ These findings underscore the necessity of integrated biomarker‐guided approaches to improve the effectiveness of ADCs in NSCLC. Currently, a significant number of ADCs aiming at NSCLC therapy are undergoing clinical trials, making them potential future treatment options. However, the clinical benefit of ADCs remains restricted to a subset of patients, demanding the discovery of reliable predictive biomarkers essential for appropriate patient stratification and individualized treatment.

In terms of cancer treatment, we are at a crossroads and unveiling the complexity of the tumor microenvironment (TME) is crucial. Proper understanding of the cellular and molecular composition of the TME is key to uncovering the mechanisms that drive tumor progression, aggressiveness and resistance or responsiveness to various available treatments (Table 1). Comprehensive characterization of the TME milieu can ultimately facilitate the discovery of novel biomarkers that guide the development of robust therapeutic strategies with improved outcomes in a greater number of patients.

**Table 1:** Cellular and noncellular components of TME and their effects on therapy response or survival.

TME constituent	Effect on TME	Models used	Method/Assay	Results
CD8^+^ T cell	Anti‐tumor	Mice & human NSCLC	Flow cytometry (FC), mIF & multispectral imaging	Based on multivariate analyses, resident memory CD8^+^ T cells demonstrate the highest functional activity and possess the strongest predictive potential for immunotherapy response.^45^
Tregs	Pro‐tumor	Mice & human NSCLC	IHC, FC & qPCR	A high number of Foxp3^+^ tumor‐infiltrating Treg cells was associated with adverse survival outcomes.^59^, ^161^
MDSCs	Pro‐tumor	Human NSCLC	RT‐PCR, FC, western blot and ELISA	CD14^+^S100A9^+^ inflammatory monocytes represent a distinct subset of MDSCs in NSCLC, causing lower T cell activity through arginase, iNOS and the IL‐13/IL‐4Rα signaling pathway. Their abundance correlates with poor chemotherapy response.^65^, ^66^
TAMs_M2 subtype	Pro‐tumor	Cell lines & human NSCLC	IHC, FC, western blot & RT‐PCR	Polarization of TAMs to M2‐like resulted in reduced phagocytosis, gefitinib‐resistant lung cancer cells and tumor xenografts.^87^
B cells	Anti‐tumor	Human NSCLC	Imaging mass cytometry (IMC)	Cellular neighborhoods with abundant B‐cells and CD4^+^ T cells were found to have improved survival.^162^
NK cells	Both Anti‐ & Pro‐tumor	Human NSCLC	mIF & GeoMx DSP	Higher expression of CD56 marker was found in the CD45^+^ stroma and could act as a prognostic and predictive marker for OS in patients undergoing PD‐1 axis inhibition therapy.^163^
CAFs	Both Anti‐ & pro‐tumor	Human NSCLC	IMC	In a cohort of over 1000 NSCLC patients, the study identified diverse CAF phenotypes associated with either favorable or unfavorable therapeutic responses. Additionally, the spatial arrangement of CAFs was found to correlate with immune cell infiltration and clinical outcomes.^164^
Hypoxia	Pro‐tumor	Cell lines and human NSCLC	IHC, cell viability assay, western blot, ELISA, RT‐PCR	Hypoxia promotes genetic instability in both tumor and endothelial cells, contributing to enhanced metastatic potential. HIF‐1α drives tumor angiogenesis primarily through upregulation of VEGF, a key angiogenic factor in lung cancer. Additionally, HIF‐1α modulates cellular energy metabolism and upregulates PD‐L1 expression in cancer cells, thereby supporting tumor progression and evading anti‐tumor immune responses.^163^, ^165^, ^166^
Metabolites	Pro‐tumor	Cell lines and human NSCLC	IHC, western blot and RT‐PCR	In the face of lung cancer, activation of metabolic pathways such as glycolysis, gluconeogenesis, fatty acid and amino acid synthesis leads to enhanced tumor proliferation and poor patient survival.^167^
Microbiome	Pro‐tumor	Human NSCLC	qPCR and metagenomic sequencing of feces and sputum	Alterations in lung and/or gut microbiota (dysbiosis) have been linked to oncogenic processes in NSCLC by influencing immune responses and metabolic pathways within the TME. Identifying specific microbial species and their related signaling mechanisms may broaden our perspective in the field of microbiome and support the development of microbiota‐targeted therapies, such as probiotics, microbial metabolites, selective antibiotics or fecal microbiota transplantation.^150^, ^151^, ^168^

CAFs, Cancer‐associated fibroblasts; HIF1α, Hypoxia‐inducible factor‐1α; iNOS, Inducible nitric oxide synthase; MDSCs, Myeloid‐derived suppressor cells; mIF, Multiplex immunofluorescence; NK, Natural Killer cells; NSCLC, Non‐small cell lung cancer; OS, overall survival; PFS, Progression free survival; TAM_M2, M2 Type tumor‐associated macrophages; VEGF, Vascular endothelial growth factor.

### Features of the tumor microenvironment

**Figure 1:**
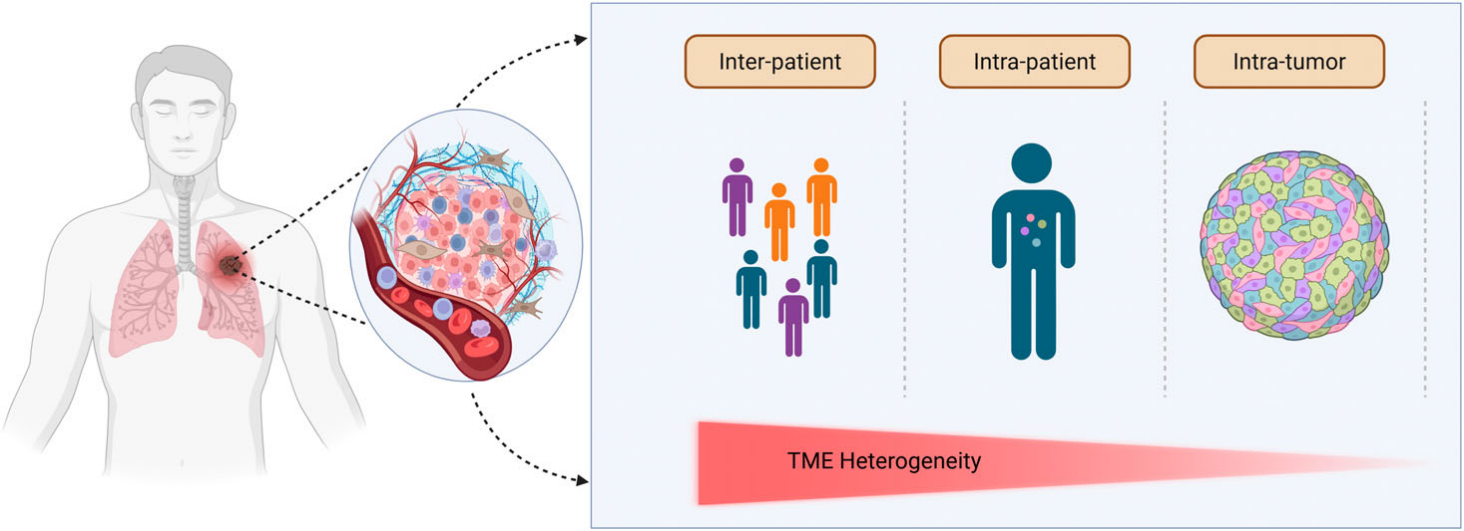
The graphical abstract depicts that the tumor microenvironment (TME) in non‐small cell lung cancer is highly heterogeneous, varying within tumors and between patients. This complexity, in turn, affects tumor growth, immune evasion and therapy resistance. Real‐time biomarker monitoring and understanding of TME dynamics are essential for improving precision medicine and patient outcomes. *Produced* using Biorender.

**Figure 2:**
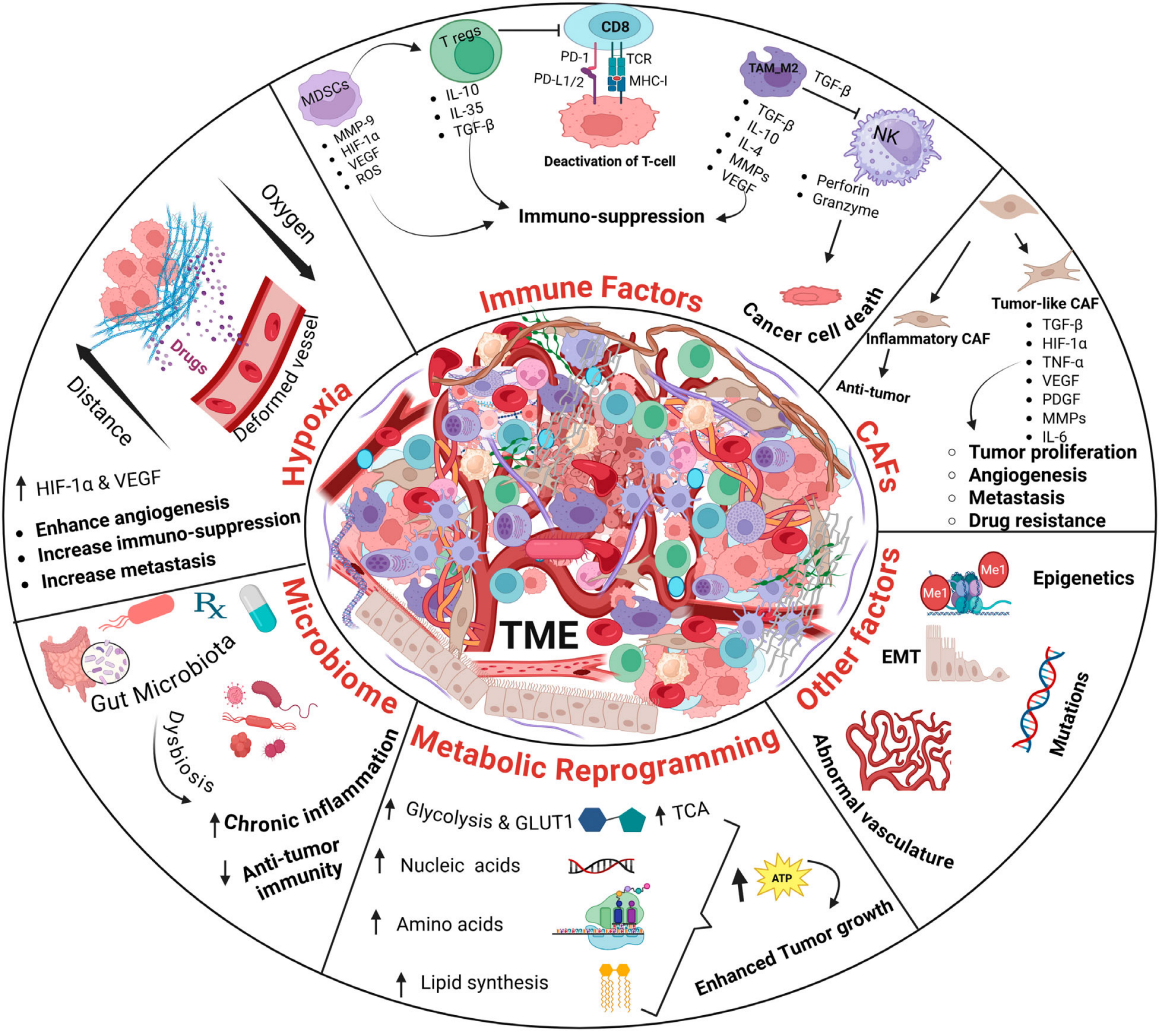
Components of the tumor microenvironment (TME). The TME is a complex eco‐system of tumor cells, immune cells, stromal cells and other noncellular elements. Overall, it is characterized by immunosuppression, hypoxia, metabolic rewiring and dysregulated vasculature leading to rapid tumor growth, immune evasion, neo‐vascularization, speedy metastasis, resistance to therapy and poor patient survival. EMT = Epithelial to Mesenchymal Transition, MMP‐9 = matrix metalloproteinase 9, GLUT1 = glucose transporter‐1, CAFs = Cancer‐associated fibroblasts, MDSCs = Myeloid‐derived suppressor cells, TAM_M2 = M2 type tumor‐associated macrophages, T regs = Regulatory T cells, NK = Natural killer cells, ROS = Reactive oxygen species. *Produced* using Biorender.

The TME is an intricate ecosystem that is constantly evolving in response to intrinsic and extrinsic factors, including genetic mutations, immune reprogramming, metabolic shifts, environmental factors, to name some.^32^ TME heterogeneity progressively increases during tumor evolution, becoming more complex across different biological levels and is conventionally classified as inter‐patient, inter‐tumoral/intra‐patient, or intra‐tumoral heterogeneity (Figure 1).^33^ The former refers to differences observed between individuals, which arise due to unique factors such as inherited genetic variants, varying mutational landscapes and environmental exposures. On the other hand, intra‐tumoral heterogeneity describes the coexistence of diverse cellular subpopulations within a single tumor, each harboring distinct genomic, epigenomic and transcriptomic characteristics.^34^, ^35^, ^36^ The TME, comprised of a diverse array of tumor and non‐tumor cells, critically influences clinical outcomes and patient survival. Therefore, a systematic understanding of the immune landscape in TME together with other cardinal features can promote the discovery of novel biomarkers and provide deeper mechanistic insight into the process of carcinogenesis, disease progression as well as design of therapeutic drugs (Figure 2).^37^, ^38^ Emerging evidence describes the crucial role of the diverse network of cellular and noncellular components of TME as outlined below.

#### Immune landscape

Cells in the human body are often subjected to carcinogenic factors or may acquire spontaneous mutations that ultimately lead to the development of cancerous cells capable of escaping detection by the immune system.^39^ Although the majority of aberrant genetic mutations are either corrected by the body's internal DNA repair mechanisms or eliminated by adaptive cellular and humoral immunity, some altered cells may still survive. These surviving cells have the potential to interfere with normal organ function or metastasize to distant areas.^39^, ^40^ In NSCLC, the tumor‐immune microenvironment is populated by a diverse array of immune cells, including CD8^+^ and CD4^+^ T cells, regulatory T cells (Tregs), myeloid‐derived suppressor cells (MDSCs), tumor‐associated macrophages (TAMs), B cells, natural killer (NK) cells and neutrophils. Collectively, these immune cell components create an immunosuppressive hub that facilitates tumor immune evasion.

Both CD8^+^ and CD4^+^ T cells belong to the lymphoid lineage and engage in the adaptive arm of the immune system. CD8^+^ T cells capture antigenic material presented via major histocompatibility complex class I (MHC‐I), which is found on the membrane of nucleated cells.^41^ Whereas CD4^+^ T cells identify peptides presented by MHC class II molecules, typically found on antigen‐presenting cells (APCs).^42^ Upon exposure to a specific antigen, activation of naïve T cells occurs, leading to their clonal proliferation and differentiation into cytotoxic T lymphocytes (CTLs).^43^ These effector T cells remain the mainstay of antitumor immunity, having the ability to release cytotoxic granules or induce cell death via the FasL‐mediated pathway to kill cancer cells.^37^, ^44^ Clinical data from NSCLC patients strongly suggest that higher densities of tissue‐resident CD8^+^ T cells correlate with improved responses to immunotherapy.^45^

Tumor‐infiltrating lymphocytes (TILs), including CD4^+^ subsets, are well‐studied in the face of cancer.^46^ Some of the CD4^+^ T cells differentiate into Tregs, constituting approximately 10% of the total CD4^+^ T cells in the circulation. These CD4^+^ Tregs express a lineage‐specific transcription factor called forkhead box P3 (FOXP3) which is necessary for cell development and immunosuppression.^47^, ^48^ Known for their suppressive function, Tregs  maintain immune homeostasis and prevent excessive inflammation and autoimmunity.^49^ A large number of tissue‐infiltrating Tregs has been associated with poorer prognosis, earlier metastatic potential, lower immunotherapy response and higher rate of recurrence in various types of solid tumors.^50^, ^51^, ^52^ Recent studies involving humans and mice have indicated that deficiency in the FOXP3 protein results in the loss of immune tolerance and hence development of an autoimmune condition involving multiple organs.^49^, ^53^ In human ovarian cancer, Ilona *et al*. found a mechanistic interaction between Tregs and macrophages where a suppressive signal is conveyed by Tregs to APCs through B7‐H4 induction (B7 family member).^54^ In another study, it was reported that Tregs can promote exhaustion and killing of T cells and APCs through enzymatic pathways,^55^, ^56^ and disrupt cellular metabolism by producing adenosine.^57^ By secreting IL‐10, IL‐35 and transforming growth factor (TGF)‐β, Tregs can inhibit antitumor immune responses.^58^ Additionally, these cells can potentially suppress cytotoxic T‐cell responses.^59^

MDSCs are crucial elements of the immune suppressive system within the TME where they inhibit host protective antitumor immunity.^60^ These cells consist of immature myeloid progenitors and lack the surface markers of mature myeloid cells.^61^ They are broadly classified as polymorphonuclear or monocytic subsets, mirroring the morphology and phenotype of neutrophils and monocytes, respectively.^61^, ^62^ Accumulating evidence suggests that MDSCs facilitate tumor growth, distant metastasis and angiogenesis by producing several biochemical factors such as TGF‐β, VEGF, basic FGF, HIF‐1, matrix metalloproteinase 9 (MMP‐9) and reactive oxygen species.^60^, ^63^, ^64^ Studies involving NSCLC patient cohorts demonstrate that myeloid cells can express l‐arginase and inducible nitric oxide synthase to suppress proliferation of CD8^+^ T cells by decreasing CD3ζ expression.^65^, ^66^ Notably, increased numbers of MDSCs (both in peripheral blood and primary lung tumor) are negatively associated with chemotherapy response and poor clinical outcome in lung tumor patients.^67^, ^68^ Through crosstalk with other TME cells, MDSCs profoundly inhibit antitumor responses. For example, these cells facilitate the emergence of cancer‐associated fibroblasts (CAFs), TAMs and Tregs in the TME. Increased levels of TGF‐β, IL‐10 and adenosine from Tregs in turn suppress the functional capacity of helper and cytotoxic T cells.^69^ In a preclinical murine model of lung cancer, Anandi *et al*. explored the therapeutic targeting of immunosuppressive MDSCs using a combination of gemcitabine and a superoxide dismutase mimetic. This treatment strategy led to enhanced activity of both effector and memory CD8^+^ T cells, and similarly improved the function of NK cells and APCs.^70^

Tumor‐associated macrophages (TAMs) are important components of the immune microenvironment network that engage in both innate and adaptive host immunity to significantly alter the fate of cancer.^71^, ^72^ The origin of these cells is primarily from circulating monocytes and/or macrophages residing in the tissues.^73^, ^74^ In adults, the main source of tissue macrophages is monocytes in peripheral blood, but longstanding tissue‐resident macrophages with self‐renewal capacity can fulfill the purpose.^75^, ^76^ In response to specific stimuli in the TME ecosystem, macrophages exist along a continuum of activation states, and their two‐tier classification as M1/M2‐like macrophages might be oversimplified.^77^, ^78^, ^79^ TAMs exert numerous supportive and inhibitory functions in lung cancer growth and dissemination via different mechanisms.^80^, ^81^, ^82^, ^83^ Pro‐inflammatory macrophages typically express markers such as CD80, CD86, HLA‐DR and inducible nitric oxide synthase (iNOS), other cytokines including IL‐1β, IL‐6 and CXCL10 that contribute to antigen presentation and enhance antitumor immunity.^73^, ^74^ In contrast, TAMs with an immunosuppressive phenotype often express different protein markers such as CD163, CD206, ARG1 and IL‐10, thereby increasing tumor‐promoting functions, including extracellular matrix (ECM) remodeling, neovascularization and suppression of antitumor immune responses.^77^ In line with this, several reports indicated that TAMs with this phenotype are highly linked to unfavorable clinical outcomes^84^, ^85^ and drug resistance in lung cancer patients.^86^, ^87^, ^88^ Furthermore, macrophages with pro‐tumorigenic behavior support tumor expansion, metastasis, neovascularization and decrease inflammation by producing many factors, such as TGF‐β, matrix metalloproteinases (MMPs) and vascular endothelial growth factors (VEGFs).^77^ Overall, TAMs exhibit a spectrum of phenotypes with distinct marker profiles and specialized functional roles, ranging from antitumor immunity to facilitating tumor progression, highlighting their plasticity and clinical influence within the TME.

Another important cellular element of the TME are tissue‐infiltrating neutrophils whose primary function is immune surveillance and inflammatory responses. Moreover, they are known to be implicated with tumorigenesis and angiogenesis.^89^ Neutrophils are believed to show both tumor promoting and/or tumor suppressing roles. On one hand, neutrophils in tumor tissue produce MMP‐9 that can degrade the ECM resulting in the generation of VEGF, subsequently stimulating the process of angiogenesis.^90^, ^91^ In models of triple‐negative breast cancer and metastatic melanoma, increased neutrophil infiltration was linked to resistance to immune checkpoint blockade therapy and accelerated tumor progression.^37^, ^92^ On the other hand, neutrophils induce the release of certain cytokines such as interferon‐gamma (IFN‐γ) from CD4^+^ cells which eventually result in differentiation of CD8^+^ T cells.^93^ In this way, IFN‐γ suppresses tumor growth by recruiting and activating the cells of both adaptive and innate immunity.^93^

#### Cancer‐associated fibroblasts

CAFs exhibit a spindle‐like elongated morphology closely resembling mesenchymal and smooth muscle cells in structure.^94^ They represent the dominant stromal cell population within the TME, where they support tumor cell growth and survival by fostering a favorable microenvironment.^95^ CAFs are central players in the TME, mediating tumor progression by remodeling the ECM and enhancing angiogenesis. While facilitating cancer cell dissemination by altering ECM architecture, they also play a structural role by producing abundant ECM components to preserve the physical integrity of the tumor.^96^ The modulatory function of CAFs in the lung cancer microenvironment is largely mediated by the secretion of a range of cytokines and growth factors that act through both autocrine and paracrine mechanisms.^97^ CAFs embedded in the TME can release several tumor‐promoting factors such as TGF‐β1, VEGF, epidermal growth factor receptor (EGFR), platelet‐derived growth factor receptors (PDGFR), hypoxia‐inducible factor 1‐alpha (HIF‐1α), tumor necrosis factor (TNF)‐α and interleukins such as IL‐6 and IL‐17A, which are critical for tumor expansion, new vessel formation, inflammation, metastasis and therapy resistance.^98^, ^99^, ^100^, ^101^, ^102^, ^103^, ^104^, ^105^, ^106^ Of note, CAFs produce MMPs, which contribute to the remodeling and breakdown of ECM, eventually converting cells within the TME to an invasive phenotype.^96^, ^106^, ^107^, ^108^, ^109^, ^110^ In contrast to the above‐stated effects, emerging human and animal studies have shown a paradoxical exacerbation of disease progression after targeting CAFs, indicating that a certain subclass of these cells might have antitumor function in the TME.^111^, ^112^

Recent single‐cell and spatial omics data reveal the presence of heterogeneous CAF subtypes with unique functional roles. In lung cancer, specific CAF populations have been identified, including myo‐fibroblastic CAFs (myCAFs) characterized by high expression of α‐SMA (ACTA2) and ECM components, inflammatory CAFs (iCAFs) that secrete cytokines such as IL‐6 and CXCL12 and antigen‐presenting CAFs (apCAFs) expressing MHC class II molecules, which may interact with T cells.^113^, ^114^, ^115^ Evolving evidence also suggests the presence of vascular‐associated CAFs and matrix‐remodeling CAFs, further highlighting the functional diversity of fibroblasts within the lung TME.^113^ Given their key roles in tumor progression and resistance to therapy, there is currently much interest in research focusing on investigating the precise CAF subsets to uncover their contribution in diagnosis and facilitate tailored therapy in lung cancer.

#### Hypoxia

Hypoxia profoundly affects the biological behavior of tumors and their response to treatment. In most solid tumors, there is rapid and uncontrolled proliferation of cells, and together with a highly abnormal tumor microvasculature, culminate in insufficient oxygen diffusion.^116^ Oxygen is vital for the generation of metabolic energy to drive many cellular processes. In the context of cancer, however, there is a disproportionate oxygen demand and supply, which aggravates tumor growth, metastasis, angiogenesis, emergence of apoptosis‐resistant cell clones, anabolic switch in central metabolism, resistance to chemo‐radiotherapy and poor clinical outcomes.^117^, ^118^ Waki and colleagues demonstrated that tumor cells in close proximity to functional blood vessels exhibited a higher proliferative capacity compared to those situated in hypoxic zones (typically 100–200 μm away from perfused vasculature or those adjacent to necrotic regions).^119^ Mounting evidence also supports that a hypoxic TME fosters genetic instability in both tumor and endothelial cells, thereby promoting metastatic dissemination.^120^, ^121^

Yang Y. *et al*. recently demonstrated that HIF‐1α plays a key role in promoting tumor neovascularization by enhancing the expression of VEGF, a crucial mediator of angiogenesis, particularly in human lung cancer.^121^ Similarly, Miao and colleagues showed that hypoxic conditions drive tumor proliferation and angiogenesis in NSCLC via the Akt/PDK1/HIF‐1α/YKL‐40 signaling cascade.^120^ HIF‐1α, a central transcriptional regulator in hypoxic conditions, also upregulates PD‐L1 expression in cancer cells, thereby dampening antitumor immune responses.^122^ It exerts broad immunomodulatory effects on both innate and adaptive immune systems, including macrophages, neutrophils, dendritic cells and lymphocytes. Likewise, hypoxia‐inducible factor‐2α (HIF‐2α) has been implicated in the regulation of nitric oxide metabolism in macrophages.^123^ Taken together, the compelling evidence linking hypoxia to cancer progression and therapy resistance emphasizes its value as a potential therapeutic target in future oncologic treatments.

#### Metabolic reprogramming

Under normal conditions, metabolism occurs in the body in an orderly fashion to meet physiological demands.^124^ In cancerous states, however, significant metabolic reprogramming occurs in the TME to ascertain the needs of rapidly growing tumor cells, which have abnormal vasculature and poor nutrition delivery.^125^ Tumor‐induced reprogramming in metabolism alters the degree and/or type of specific metabolites within and outside cells and encourages tumor proliferation by affecting gene expression, cell activation, and composition of the TME.^126^ During tumorigenesis, the key metabolites that are subject to metabolic change include glucose, glutamine and lipids, and targeting these pathways suppresses tumor growth and promotes programmed cell death.^126^ Tumor cells utilize large amounts of glucose, even when enough oxygen is available, to produce lactic acid as a byproduct. Surprisingly, the energy generated via this process is much lower (2 mol ATP/mol glucose) than would be produced by normal cells' glycolysis machinery ATP (34 mol ATP/mol glucose).^127^ Nevertheless, tumor cells adopt certain biosynthetic pathways to produce various intermediate metabolites that can be used as building blocks for amino acids, lipids, nucleotides and reduced nicotinamide adenine dinucleotide phosphate (NADPH) to ensure sustained growth. For instance, RNA and NADPH are generated through the pentose phosphate pathway,^128^ protein glycosylation takes place via the hexosamine pathway,^129^ and amino acids are formed from the serine biosynthesis pathway.^130^

Alterations in glucose metabolism significantly influence tumor progression and contribute to resistance against various therapies.^126^ A group of carrier proteins called glucose transporters (GLUTs) enables the movement of glucose across the cell membrane.^126^ In one study, GLUT‐1 in A549 lung cancer cells was irreversibly blocked by WZB117 that resulting in reduced glucose uptake, suppressed cell cycle progression and increased cancer cell death.^131^ Moreover, the activation of several glycolytic enzymes in lung cancer has been linked to therapy resistance and unfavorable survival outcomes. Elevated expression of enzymes such as hexokinase 2, phosphofructokinase 1 (PFK1), the less active isoform pyruvate kinase M2 (PKM2) and lactate dehydrogenase (LDH) has correlated with accelerated tumor proliferation and exacerbated resistance to therapy.^132^ Among amino acids, glutamine plays a major metabolic role by acting as a source of carbon and nitrogen for rapidly growing tumor cells. It also helps maintain redox homeostasis by regulating intracellular reactive oxygen species, which in turn, prevents oxidative stress and increases the survival of tumor cells.^133^ Unlike healthy cells, where *de novo* synthesis of lipids is limited to specific organs (such as fat tissue, liver and lactating breast), cancer cells possess the ability to synthesize excessive amounts of fatty acids for structural and functional purposes.^134^ Specifically, in times of low plasma glucose, tumor cells degrade fatty acids to generate enough ATP via the tricarboxylic acid, ultimately meeting the excess energy demand of tumor cells.^134^, ^135^ In summary, metabolic reprogramming in the TME is a complex process that involves dynamic interaction between tumor cells, stromal cells and immune cells. Hence, proper understanding of these metabolic alterations grants opportunities for therapeutic manipulation of specific metabolites that would open an avenue for cancer treatment.

#### TME's vasculature

The tumor vasculature in NSCLC undergoes profound structural and functional remodeling driven by dysregulated VEGF/VEGFR signaling and other angiogenic pathways.^136^ These alterations cause tortuosity and hyperpermeability of vessels covered by abnormal pericyte, resulting in impaired perfusion and marked intratumoral hypoxia.^136^ Tumor endothelial cells adopt distinct transcriptional and immunomodulatory phenotypes, altering adhesion molecule expression, antigen‐presenting capacity and checkpoint ligand profiles while fostering recruitment of MDSCs and Tregs.^137^ Hypoxia further reinforces immunosuppression via HIF‐dependent programs that limit effector T‐cell infiltration and support exhausted or dysfunctional immune states.^123^ Spatial and single‐cell profiling studies demonstrate that perivascular niches enriched in M2‐like macrophages, Tregs and exhausted CD8^+^ T cells act as focal points of immune exclusion, linking vascular pathology directly to therapeutic resistance.^138^ These insights emphasize that angiogenesis in NSCLC is not merely a growth‐supportive process but a central mechanism shaping immune surveillance and TME composition.

Understanding these vascular and immune abnormalities provides a strong mechanistic basis for combining anti‐angiogenic therapies with ICIs in NSCLC. Anti‐VEGF agents can transiently normalize tumor vasculature, improve perfusion and facilitate effector T‐cell infiltration, while simultaneously reversing VEGF‐mediated suppression of dendritic‐cell maturation and reducing Treg/MDSC accumulation.^136^, ^139^ Clinically, the synergy between VEGF‐pathway inhibition and immunotherapy is best demonstrated in a phase 3 clinical trial (IMpower150), where adding bevacizumab to atezolizumab plus chemotherapy produced significant improvements in progression‐free and overall survival, including in challenging subgroups such as EGFR‐mutated tumors following their targeted therapy.^140^ Additional studies with bevacizumab, ramucirumab and VEGFR/MET/AXL‐inhibiting tyrosine‐kinase inhibitors support the principle that targeting tumor vasculature can reshape the TME to enhance responsiveness to ICIs and overcome primary or adaptive resistance.^141^ Collectively, these mechanistic and clinical data position vascular–immune crosstalk as a key therapeutic axis in NSCLC, complementing and strengthening immunotherapy efficacy.

#### Crosstalk between microbiota and TME

Microbiota refers to a community of commensal microbes whose presence in the mucosal and epithelial barriers is highly influential in both health and disease states.^142^ Disruptions to this ecological niche of microbiota leads to changes in its homeostatic composition and function. For instance, provision of broad‐spectrum antibiotics or chemotherapy results in the replacement of normal gut flora by more pathogenic strains, subsequently limiting the production of microbiota‐derived metabolites, which are critical for immune cells' development and maintenance.^143^

Recent research has highlighted the role of the microbiome in tumorigenesis, cancer progression and therapy response.^142^ In particular, the close link between the microbiome and the TME has become a focal point of investigation, revealing complex mechanisms that shape tumor pathobiology. The gut microbiota, in particular, exerts broad systemic effects on host immunity and metabolism, thereby indirectly shaping the TME.^142^ Microbial products such as short‐chain fatty acids (SCFAs), bile acids and polyamines can enter the circulation and exert either direct or indirect effects on tumor and immune cells within the TME.^144^ For example, SCFAs like butyrate have been found to enhance immunity against cancer cells by stimulating both regulatory T cells and cytotoxic T cells.^144^ Conversely, certain microbial metabolites can promote tumor growth by inducing inflammation or suppressing immune responses.^145^

The microbiome also influences TME through its impact on systemic immunity. Gut microbiota can modulate the differentiation and migration of several immune cells such as T cells, dendritic cells and macrophages to the TME; hence, their function is altered accordingly.^146^ There is a strong connection between imbalance in the microbiota niche (dysbiosis) and the occurrence of persistent inflammation and immuno‐suppression, both of which are hallmarks of cancer. For instance, specific bacterial strains such as *Fusobacterium nucleatum* have been associated with colorectal cancer by promoting inflammation and creating an immunosuppressive TME.^142^ Several clinical studies involving patients with NSCLC, melanoma, renal cell carcinoma and urothelial carcinoma have revealed that the abundance of gut microbiota correlates with response to immune checkpoint inhibitors, based on analyses of fecal microbiota.^147^

Beyond systemic effects, local microbiomes at tumor sites can directly shape the tumor niche. Tumors arising in organs like the lungs, gastrointestinal tract and skin harbor distinct microbial ecosystems, which can influence local tumor behavior. In colorectal cancer, for example, intra‐tumoral bacteria can directly interact with tumor cells and immune cells to modulate the NF‐κB and Wnt/β‐catenin signaling pathways, processes which are central to cancer progression.^148^, ^149^ Similarly, in pancreatic cancer, the resident microbiome has depressed immune‐surveillance via recruitment of MDSCs and lowering the activity of T cells. Cameron SJ. *et al*. used metagenomic sequencing of the microbiome using sputum samples, and their findings suggested potential bacterial biomarkers for lung cancer.^150^, ^151^ Despite the growing understanding of the microbiome–TME axis, several challenges remain. First, as immunotherapy works by reinvigorating the immune system to eliminate cancer cells, there is reciprocal interaction between immunotherapy and the microbiome. Therefore, while response to immunotherapy is affected by the microbiome, the immunotherapy itself might lead to unwanted consequences on the microbiome.^142^ Second, the complexity and variability of microbial communities across individuals make it difficult to establish universal therapeutic targets.^142^ Moreover, the mechanisms underlying microbiome‐TME interactions are not fully elucidated, necessitating further research.^142^ In this case, cutting‐edge technologies such as multi‐omics approaches and single‐cell sequencing will be instrumental in unraveling these complexities.

In conclusion, the microbiome is a key modulator of the TME, influencing tumor biology and treatment response through systemic and local mechanisms. Exploring the interplay between the microbiome and TME offers significant promise for improving cancer treatment. However, achieving this will require a deeper understanding of the intricate communication between microbial communities, host cells and the TME.^147^ As research continues to evolve, microbiome‐targeted therapies, either alone or combined with immunotherapy, may become essential components of personalized cancer care, bringing renewed hope to patients.

## IMMUNE‐BASED THERAPY IN NSCLC

The clinical utility of immunotherapy, particularly ICIs, has significantly transformed cancer care by leveraging the body's immune system to identify and attack malignant cells.^152^, ^153^ Unlike traditional therapies (chemotherapy and radiation) that directly attack cancer cells, immunotherapy enhances immune recognition and response, offering the potential for durable remission in previously untreatable cancers.^154^ There are many immune‐based treatment approaches, such as ICIs, vaccine‐ and cell‐based immunotherapies, all aiming at establishing or promoting effective immune reactions against cancer cells.^153^ Among the immunotherapy approaches, ICIs are extensively used in clinical settings owing to their proven survival benefits and improvement in quality of life as compared to chemotherapy. They are generally well‐tolerated and effective, which makes them a suitable therapeutic option for selected NSCLC patients.^155^

At the molecular level, ICIs exploit the presence of cell surface markers, such as PD‐L1/PD‐1 and CTLA‐4, to unleash the body's immunity against tumors.^152^ The use of ICIs in clinical settings has remarkably altered the treatment landscape in NSCLC, with established roles in cases that are unresectable, locally advanced or suitable for perioperative management. After confirmation of its efficacy, ipilimumab, a CTLA‐4 blocker, was the first ICI to be approved by the FDA in 2011 for the treatment of patients with advanced melanoma.^152^ Even though platinum‐based chemotherapy dominates as the current first‐line treatment for lung cancer, developing novel combination therapeutic strategies is vital for promoting anti‐tumor responses and enhancing survival outcomes.^11^ Dual checkpoint inhibition (targeting two checkpoints simultaneously) has emerged as a promising strategy, demonstrating higher response rates than monotherapy, albeit with increased toxicity. The integration of dual blockade immunotherapy along with engineered bispecific antibodies is another approach that may further potentiate immune activity.^11^ Initially, the clinical application of anti‐PD‐L1/PD‐1 immunotherapy was to serve as second line monotherapy. However, there has been recent progress in maximizing immunotherapy by combining it with chemotherapy in the first line setting.^156^ The combination of pembrolizumab (an anti‐PD‐1 ICI) with platinum‐based chemotherapy has shown clinical benefits and has been approved based on KEYNOTE‐407^157^ and KEYNOTE‐189.^158^ In addition, other ICI agents atezolizumab and cemiplimab were combined with chemotherapy and have led to regulatory approvals as first‐line therapy for advanced NSCLCs without driver mutations.^159^, ^160^

Overall, immunotherapy has revolutionized oncology, offering curative potential for some patients. Despite existing challenges such as treatment resistance, drug‐related adverse effects and financial burdens, ongoing advances in biomarker development, rational drug combinations and personalized treatment strategies are expanding their therapeutic potential. Future progress hinges on multidisciplinary collaboration, equitable access and deeper insights into immune‐tumor dynamics.

## ROLE OF MULTI‐OMICS PROFILING IN A GEOSPATIAL CONTEXT

Cancer is a highly heterogeneous disease, with significant variability in genetic, epigenetic and microenvironmental factors among cells within the TME.^32^ The rapid technological advancement in the omics field has enabled researchers to quantify biomolecules, including DNA, RNA, protein and metabolites at different scales.^176^ To date, these technologies have been applied for a multitude of purposes.^176^, ^177^ First, they are used as a validation tool to determine the identity of newly discovered cells that were initially revealed by other methods (e.g. single‐cell sequencing).^177^ Second, they give information on the localization of captured cells and hence allow cell‐to‐cell or neighborhood analysis without disrupting tissue integrity.^177^ Third, previously hidden mechanistic molecular interaction between ligand and receptor can be elucidated.^177^

**Figure 3:**
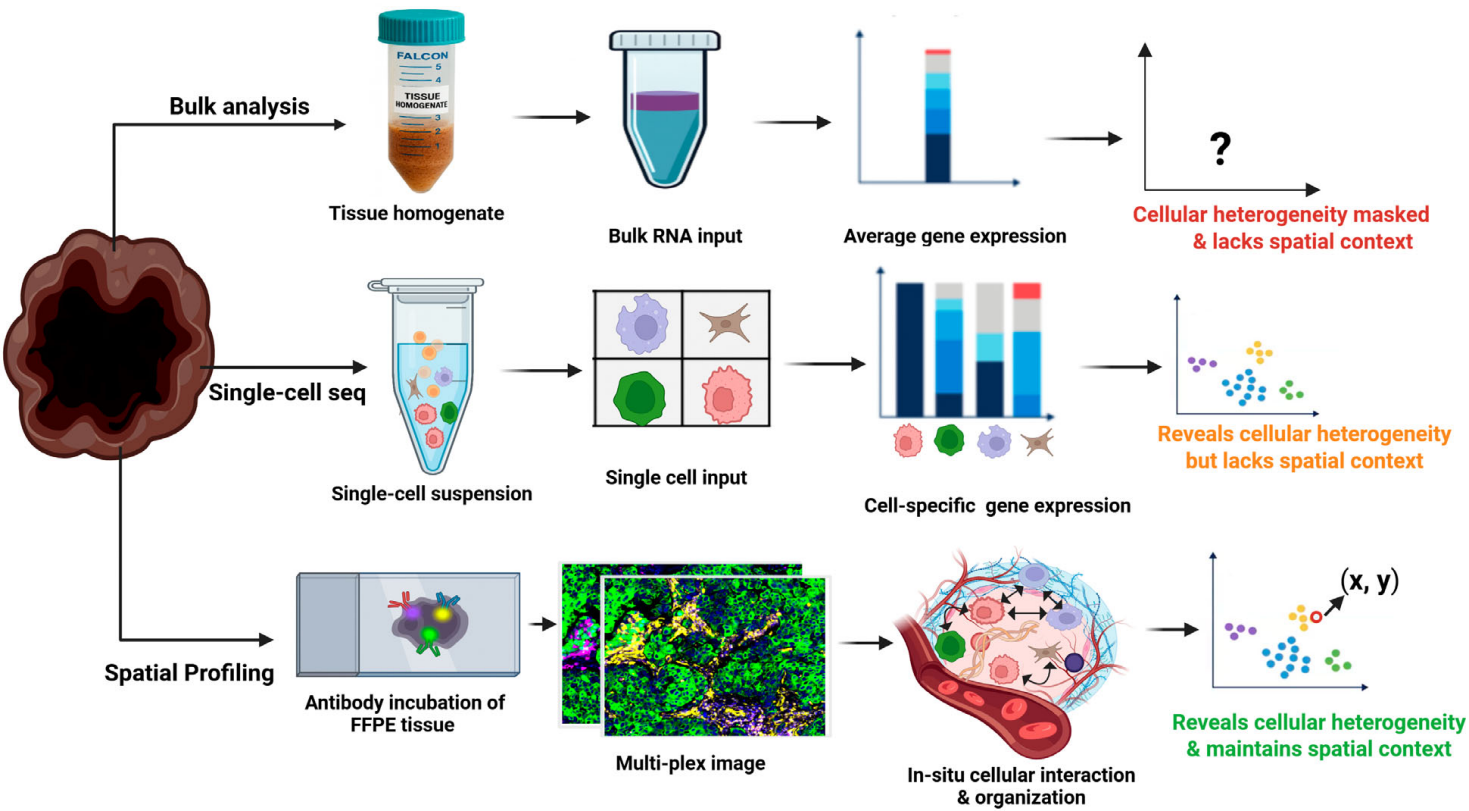
Comparative overview of bulk, single‐cell and spatial profiling approaches. Bulk profiling methods provide sample‐level average molecular readouts across heterogeneous tissues, effectively capturing dominant signals while masking underlying cellular diversity. Single‐cell profiling enables high‐resolution delineation of individual cell identities and functional states across multiple molecular layers, yet typically requires tissue dissociation, resulting in the loss of spatial context and native cell–cell relationships. Spatial profiling technologies overcome these limitations by preserving tissue architecture, enabling *in situ* multi‐omic characterization of cellular heterogeneity and revealing the spatial organization and interactions that underpin tissue function and disease biology. *Produced* using Biorender.

Unlike the spatial methods, bulk and single‐cell genomic sequencing approaches describe dissociated cells in a sample mixture with loss of their geospatial organization (Figure 3). But they are still capable of capturing diverse molecular and cellular identity at the genomic scale level, a feature that has led to a vast array of exciting biological breakthroughs.^178^ Nowadays, the emergence of spatially resolved, multiplex genomic, transcriptomic and proteomic profiling techniques is gaining popularity, yielding a systematic characterization of molecules and cells throughout the tissue space.^178^ Although individual spatial platforms, such as spatial transcriptomics, provide robust datasets, integrating multiple omic layers is essential to gain a more nuanced understanding of cell identity and function.^178^, ^179^ In essence, integration of all spatial omics data (transcriptomic, proteomic or epigenomic) with other methods, such as single‐cell sequencing, is warranted to overcome the limitations of any single approach and maximize the confidence in discoveries when multiple technologies converge on similar results.^177^, ^178^, ^179^ Several spatial profiling methods have shown considerable promise in both cancer diagnostics and therapeutic decision‐making, particularly by enabling high‐resolution analysis of individual tumor cells within their native microenvironment (Table 2).^180^ Specifically, in the era of immunotherapy, spatial technologies have been useful in dissecting cells of the TME before and/or after ICI therapy, identifying predictive biomarkers of resistance or response, evaluating effector function of immune cells and discovering potential immune checkpoints.^181^

**Table 2:** Overview of selected studies that utilized spatial tools to identify prognostic or predictive biomarkers in NSCLC.

Spatial feature studied	Technology platform	Year of study	Molecular Plex	Key findings
Spatial immune landscape of LUAD & LUSC TME	Imaging mass cytometry	2025	35	CD163^−^ macrophages (M1‐like, pro‐inflammatory) exhibited increased interactions with regulatory T cells in LUAD, with both cell types being more prevalent in LUAD than in LUSC.^91^
Tumor‐Stroma Immune cell interactions	PhenoCycler‐ Fusion	2024	25	The co‐localization of macrophages (*P* = 0.003) and effector CD4^+^ T cells (*P* = 0.01) within mixed tumor niche and CD8^+^ T cells within HLADR^+^ tumor neighborhoods (*P* = 0.03), was significantly associated with favorable clinical outcomes.^169^
CAF phenotypes and their interaction with immune cells	Imaging mass cytometry	2024	48	The study revealed unique phenotypic and spatial characteristics of CAF subtypes linked to clinical outcomes in NSCLC. Specifically, tumor‐like CAFs (tCAFs) and hypoxic tCAFs were more prevalent in cases with metastatic disease, post‐chemotherapy relapse and reduced overall survival.^164^
Tumor‐Stroma Immune cell interactions	Nanostring GeoMx DSP	2024	78	The researchers showed that CD56^+^ in the stromal area resulted in improved survival (HR = 0.172, *P* = 0.001), while B7.H3 in the tumor (HR = 1.72, *P* = 0.008) was linked to poorer survival.^170^
Immune lineage and activation in TME	Imaging mass cytometry	2023	35	The study analyzed tumor and immune features in lung adenocarcinoma samples from 416 patients across five histological subtypes. B‐cell and CD4^+^ T cell enriched neighborhoods exhibited better clinical response.^162^
Tumor‐Immune compartment analysis	Nanostring GeoMx DSP	2022	71	Intra‐tumoral CD44 expression as compared to the immune regions (panCK^−^/CD45^+^) was significantly correlated with better clinical outcomes (OR = 1.22, *P* = 0.018) and longer progression‐free survival under PD‐1 axis blockade when stratified by the highest tertile (multivariate HR = 0.62, *P* = 0.03).^171^
Tumor‐Stroma Immune cell infiltration	Imaging mass cytometry	2022	26	Developed a spatially resolved maps (SpatialVizScore) using multiplexed markers and created a three‐tier classification of patients' tumors including immune‐inflamed, immune‐suppressed and immune‐cold states.^172^
Infiltration of TAMs to the TME	Nanostring GeoMx DSP	2022	44	Increased TAMs in NSCLC TME are associated with resistance to immunotherapy regardless of the level of PD‐L1 expression and driven by upregulation of specific genes (CD27, ITGAM and CCL5) within the tumor compartment.^173^
Spatial profiling of TILs	Multiplexed IF	2022	5	TILs were predominantly found in stromal regions adjacent to clusters of tumor cells, with CD8^+^ T cells being the highest subtype. A higher number of stromal CD8^+^ cytotoxic T cells was correlated with improved survival outcomes, particularly those with high PD‐L1 expression.^174^
Tumor‐Stroma‐Normal tissue comparison	Nanostring GeoMx DSP	2020	53	The presence of CD3‐expressing cells in tumor areas resulted in better treatment outcomes.^175^

CAFs, Cancer‐associated fibroblasts; DSP, Digital spatial profiling; IF, Immunofluorescence; TAMs, Tumor‐associated macrophages; TILs, Tumor‐infiltrating lymphocytes.

High‐plex spatial imaging technologies have markedly expanded our ability to dissect the TME of NSCLC, with CODEX representing one of the most powerful platforms for protein‐level, single‐cell resolved tissue mapping.^169^, ^182^ CODEX enables simultaneous quantification of dozens of protein markers across intact tumor sections, allowing precise delineation of immune, stromal and malignant cell states while preserving their spatial relationships. Using CODEX, key TME hallmarks can be interrogated in situ, including spatial organization of cytotoxic, helper and regulatory T‐cell subsets; B cell and tertiary lymphoid structures; diverse macrophage and dendritic cell phenotypes; CAF niches; and gradients related to proliferation, metabolic stress and hypoxia.^115^, ^169^, ^183^ These unique spatial ecosystems closely correlate with clinical outcomes. CODEX studies in NSCLC have shown, for example, that the degree of spatial intermixing between CD8^+^ T cells and PD‐L1 expressing tumor or myeloid cells, the density and maturity of tertiary lymphoid structures, and the emergence of myeloid‐rich suppressive neighborhoods outperform bulk immune‐density metrics in predicting therapeutic response and overall survival.^169^, ^184^ While complementary platforms such as IMC, Multiplexed Ion Beam Imaging (MIBI), multiplex IF and spatial transcriptomics (e.g. Visium, GeoMx DSP, CosMx Spatial Molecular Imaging) offer broader molecular or transcriptomic coverage, CODEX remains uniquely suited for high‐resolution protein phenotyping across large regions of interest, enabling nuanced reconstruction of spatially organized tumor‐immune interactions.^185^

Monkman *et al*. have conducted several studies on formalin‐fixed paraffin‐embedded (FFPE) samples of various human cancers by spatially mapping the TME.^169^, ^170^, ^175^, ^186^ In one study using high‐plex Digital Spatial Profiling (DSP), they analyzed tissue microarrays from NSCLC patients and reported enrichment of immune markers such as CD27, CD3, CD4, CD44, CD45, CD45RO, CD68, CD163 and VISTA within the TME compared to tumor regions. Using univariate Cox regression, they found that the expression of CD3 (HR: 0.5, *P* = 0.018), CD34 (HR: 0.53, *P* = 0.004) and ICOS (HR: 0.6, *P* = 0.047) were associated with increased overall survival.^175^ In another study, the above researchers evaluated transcriptomic and proteomic profiles of ICI‐treated advanced NSCLC (*n* = 41) by using DSP and multiplex IHC. Here, their findings disclosed difference in cellular interaction and expression level of specific genes within the TME of cohorts. Interestingly, they found concurrently increased expression of IL2 receptor alpha (*P* = 0.028) and IL2 mRNA (*P* = 0.001) in the tumoral and stromal compartments, respectively.^182^ Moreover, Kulasinghe *et al*. applied highly multiplexed CODEX imaging (Akoya Biosciences, US) to investigate the spatial and phenotypic attributes of the TME in NSCLC patients treated with ICIs.^169^ In the resistant group, Tregs were more frequently adjacent to monocytes (*P* = 0.009) and CD8^+^ T cells (*P* = 0.009). Conversely, responders had greater macrophage proximity to HLADR^+^ tumor cells (*P* = 0.01). Cellular neighborhood analysis further showed that macrophages (*P* = 0.003) tumorigenesis and effector CD4^+^ T cells (*P* = 0.01) in mixed tumor areas, as well as CD8^+^ T cells (*P* = 0.03) in HLADR^+^ regions, were associated with favorable clinical outcomes.^169^ Additionally, recent studies by Rimm *et al*. analyzed spatial transcriptomic and proteomic profiles of NSCLC cohorts (before & after treatment, respectively) to identify biomarkers of ICI response.^171^, ^187^ Geospatial transcriptomic analysis using DSP in pretreatment samples of 56 patients indicated that the protein biomarker CD66b in the CD45^+^ CD68 stromal immune regions revealed lower overall survival (hazard ratio [HR] 1.31, *P* = 0.016).^187^ Likewise, in a study involving ICI treated NSCLC patients, the above researchers employed DSP to spatially resolve proteomic profiling of the TME and discovered that the biomarker CD44 resulted in better response to ant‐PD‐1 axis therapy which might guide to the development of improved immunotherapy strategies.^171^

Despite their transformative insights, spatial technologies (CODEX included) have inherent technical and analytical limitations. Imaging‐based platforms depend heavily on tissue quality, antibody validation, staining reproducibility and robust segmentation algorithms; panel size, although high‐plex, remains constrained relative to transcriptome‐wide in situ hybridization approaches.^185^, ^188^ Analytical variability across pipelines spans from cell calling to neighborhood definitions that limit reproducibility and cross‐cohort comparability.^188^ Spatial transcriptomic platforms provide greater molecular breadth but at the cost of lower single‐cell resolution or region‐of‐interest‐based sampling.^188^, ^189^ Nevertheless, integration of CODEX‐derived spatial proteomics with genomic, transcriptomic and clinical datasets has advanced understanding of NSCLC biology by mapping immune‐excluded versus immune‐inflamed tumor states, identifying perivascular and stromal suppressive niches underpinning ICI resistance and defining tumor‐ and stroma‐specific signatures associated with survival.^185^

In summary, CODEX and complementary spatial modalities have immensely increased our knowledge on TME's complexity by preserving spatial relationships and contextualizing molecular and cellular profiles.^190^ These approaches have deepened insights into treatment resistance, tumor evolution and heterogeneity.^190^ Information gleaned through these integrated technologies provides inroads into more accurate cancer diagnostics, personalized treatment strategies and improved patient outcomes. Despite current challenges, continuous innovations in bioinformatics, artificial intelligence and cost‐effective sequencing methods will likely accelerate their integration into routine oncology practice.

## CONCLUSION

The dynamic nature of the TME in NSCLC has both challenges and opportunities. Looking at the current situation of immune‐based cancer therapeutics, there are a few critical questions worth raising: (1) How can we better stratify patients for immunotherapy, ensuring broader inclusion while optimizing combination treatment strategies? (2) What are the underlying biological mechanisms responsible for resistance to immune checkpoint inhibitors? (3) Can we identify additional predictive biomarkers and immune checkpoint targets beyond those currently available, such as PD‐L1/PD‐1 and CTLA‐4? (4) Are existing preclinical models adequate for exploring the molecular basis of resistance and discovering novel therapeutic targets? (5) How can we maximize the therapeutic efficacy of ICIs while minimizing their immune‐related toxicities? (6) Can emerging computational and artificial intelligence‐driven approaches enable the integration of high‐dimensional datasets such as single‐cell and spatial‐omics data to uncover previously unrecognized cellular interactions, identify novel therapeutic targets and refine predictive biomarkers for immunotherapy response in NSCLC?

Achieving a deeper understanding of tumor evolution at the single‐cell level, especially through appropriate preclinical modeling, holds the potential to revolutionize personalized cancer therapy by enhancing treatment responses and overcoming resistance. Advanced technological platforms, including single‐cell sequencing and high‐resolution spatial multi‐omics, are proving invaluable for unraveling the complexity and heterogeneity of the TME across cancer subtypes. These insights are expected to facilitate the development of new therapeutic strategies and improve patient outcomes. While spatial profiling techniques for target discovery are still developing, multiplexed spatial analysis of the TME offers promise in drug development and clinical trial design by revealing intricate cell–cell interactions that contribute to cancer progression and resistance mechanisms. Looking ahead, research aims to expand the therapeutic reach of immunotherapy by introducing novel agents, refining biomarker‐driven strategies and exploring effective combinations while addressing the persistent issues of resistance and toxicity. Though challenges such as cost and accessibility remain, continued scientific progress is paving the way toward fully integrated, stage‐independent personalized oncology.

## AUTHOR CONTRIBUTIONS

Concept: KSE, AK. Writing and critical review: all authors.

## CONFLICT OF INTEREST

Arutha Kulasinghe is on the Scientific Advisory Board for Omapix Solutions, Predxbio, Molecular Instruments and Visiopharm. All other authors declare no financial or nonfinancial competing interests.

## Data Availability

Data sharing not applicable to this article as no datasets were generated or analysed during the current study.
